# Effectiveness and Cost-Effectiveness of a Stepped Model of Care for Musculoskeletal Disorders: Protocol for a Multiarm Randomized Controlled Trial (Edu-First Trial)

**DOI:** 10.2196/77574

**Published:** 2025-11-19

**Authors:** Jean-Sébastien Roy, Hugo Massé-Alarie, Marc-Olivier Dubé, Anne Marie Pinard, Martin Lamontagne, Gisela Sole, Jean Tittley, Maude Laberge, Frédérique Dupuis, Félix Fiset, Ildephonse Nduwimana, Eric McArthur, François Desmeules

**Affiliations:** 1 Faculty of Medicine Université Laval Quebec City, QC Canada; 2 Centre for Interdisciplinary Research in Rehabilitation and Social Integration (Cirris) Quebec City, QC Canada; 3 La Trobe Sport and Exercise Medicine Research Center La Trobe University Melbourne Australia; 4 Faculty of Medicine University of Montreal Montréal, QC Canada; 5 School of Physiotherapy University of Otago Dunedin New Zealand; 6 VITAM Quebec City, QC Canada; 7 CHU de Québec-Université Laval Research Center Quebec City, QC Canada; 8 London Health Sciences Centre Research Institute London, ON Canada; 9 Maisonneuve-Rosemont Hospital Research Center Montreal, QC Canada

**Keywords:** education, cost-effectiveness, rehabilitation, shoulder pain, low back pain, anterior knee pain, neck pain.

## Abstract

**Background:**

Musculoskeletal disorders (MSKDs) are a leading cause of pain and disability, placing a substantial burden on health care systems. Optimizing resource use through innovative interventions is essential. Evidence from randomized controlled trials suggests that not all individuals with MSKDs require ongoing follow-up with a health care provider; for many, education alone is sufficient for symptom resolution. A stepped care model, which prioritizes patient education as a first-line intervention and reserves usual care for those with persistent symptoms, may enhance health care efficiency and reduce costs.

**Objective:**

The primary objective of this randomized controlled trial is to evaluate the effectiveness of a stepped care model compared to the 2 most common approaches for managing MSKDs: usual medical care and usual rehabilitation care. A secondary objective is to assess cost-effectiveness.

**Methods:**

This pragmatic, noninferiority, multiarm, parallel-group randomized controlled trial will enroll 369 adults with MSKDs, randomly assigned to one of three 12-week intervention groups: stepped care, usual medical care (physician-led), or usual rehabilitation care (physiotherapist-led). Participants in the stepped care group will first complete a 6-week education program. Those with persistent symptoms after 6 weeks will receive rehabilitation interventions, while participants whose symptoms have resolved will receive no further intervention. The primary outcome is functional limitations at 24 weeks. Secondary outcomes include pain severity, health-related quality of life, pain-related fear, and pain self-efficacy, assessed at baseline and at 6, 12, and 24 weeks. Linear mixed models will be used for group comparisons, and incremental cost-effectiveness analyses will evaluate cost-effectiveness. The ethics committee of the CIUSSS-CN approved the project (#2024-2982). Findings will be shared through clinical and community platforms, peer-reviewed publications, and conference presentations.

**Results:**

The Edu-First trial is funded by a project grant from the Canadian Institutes of Health Research (grant #495615). Recruitment began on January 31, 2025. As of September 2025, a total of 65 participants have been enrolled. Recruitment is expected to continue for up to 3 years, targeting approximately 10 new participants per month, and is anticipated to be completed by Winter 2028.

**Conclusions:**

We anticipate that the stepped care model will be noninferior to usual medical care and usual rehabilitation care in terms of treatment effectiveness. Furthermore, it is expected to be cost-effective by reducing reliance on expensive resources, such as provider consultations and medical investigations. By emphasizing education and self-management as the initial approach, the stepped care model may enhance access to care without compromising quality, while empowering patients to actively manage their condition. Findings from this study could inform systemic changes in MSKD care delivery, improving treatment accessibility and reducing the average cost per care episode.

**Trial Registration:**

ClinicalTrial.gov NCT06832852; https://clinicaltrials.gov/ct2/show/NCT06832852

**International Registered Report Identifier (IRRID):**

DERR1-10.2196/77574

## Introduction

Musculoskeletal disorders (MSKDs) have become a global epidemic, with their prevalence steadily rising over the past decades [1]. This trend is expected to persist due to aging populations and ongoing exposure to environmental and occupational risk factors [1,2]. MSKDs significantly impact individuals by limiting activities and restricting participation while also placing a heavy burden on society through work absenteeism, disability pensions, early retirement, and increasing demands for social support [3,4]. While some individuals experience short-term symptoms, others endure persistent or recurrent pain, leading to impaired daily functioning and substantial health care costs [5-7]. MSKDs are among the most common reasons for seeking primary care, with up to 20% of adults consulting their family physician for an MSKD-related issue annually [8]. Ensuring timely and appropriate care for individuals with MSKDs remains a challenge, even in countries with universal health care [9].

A major barrier to health care access for individuals with MSKDs is prolonged wait times [10-12]. In addition, significant gaps and disparities in access to publicly funded health care persist across several countries [11,13-15]. A systematic review reported significant declines in health-related quality of life and psychological well-being among those experiencing treatment delays [16]. Longer wait times have also been associated with increased health care use, delays, or restrictions in work participation [17]. To address these challenges, health care services must be redesigned to reduce wait times and promote more equitable access. A systematic review examining strategies to reduce waiting times in outpatient rehabilitation services for adults with MSKDs found that several service redesign approaches—such as referral management strategies and alternative models of care—effectively decreased waiting times while better meeting patients’ needs [18]. Given the negative consequences of prolonged delays, implementing efficient and effective strategies to improve timely access to care for individuals with MSKDs is essential.

In 2 randomized controlled trials, we demonstrated that not all patients with chronic MSKDs require close follow-up by a health care provider, as for a large proportion, patient education alone was sufficient to achieve significant improvement or even resolution of their condition [19,20]. As a first-line approach in primary care, patient education is cost-effective, widely accessible, and adaptable [11,21], and in some cases, it is as effective as usual care [22,23]. It can be delivered by various health care providers in multiple formats, including self-administered options, depending on the context and individual needs [24]. The primary goals of education are to improve patients’ understanding of their condition, dispel myths and misconceptions, promote healthy lifestyle choices, and optimize self-management [25-27]. A better understanding of the condition and its psychological factors enables individuals to manage their symptoms more effectively [28,29]. As health care costs rise, a stepped care model—where patient education and advice are offered first, with usual care provided only to those whose symptoms persist—could improve access to health care by reducing the number of visits with a health care provider without compromising quality of care.

The primary objective of this randomized controlled trial is to determine whether a stepped care model is as effective as the 2 most common care models—usual medical care (led by a family physician) and usual rehabilitation care (led by a physiotherapist)—in individuals with MSKDs at 24 weeks post allocation. The primary outcome will be functional limitations at 24 weeks. Secondary outcomes will include functional limitations at 6 and 12 weeks, as well as pain intensity, health-related quality of life, region-specific symptoms and functional limitations, pain-related fear, pain catastrophizing, pain self-efficacy, and satisfaction with care at 6, 12, and 24 weeks. The secondary objective is to investigate the cost-effectiveness of a stepped care model compared to usual medical care and usual rehabilitation care for managing MSKDs in Canadian adults over a 24-week period. We hypothesize that the stepped care model will be noninferior to usual medical care and usual rehabilitation care in terms of effectiveness. In addition, we expect health care costs to be significantly lower for the stepped care model due to a reduction in the use of costly resources, such as consultations with health care providers and medical investigations (eg, laboratory tests and medical imaging).

## Methods

### Study Design

**Figure 1:**
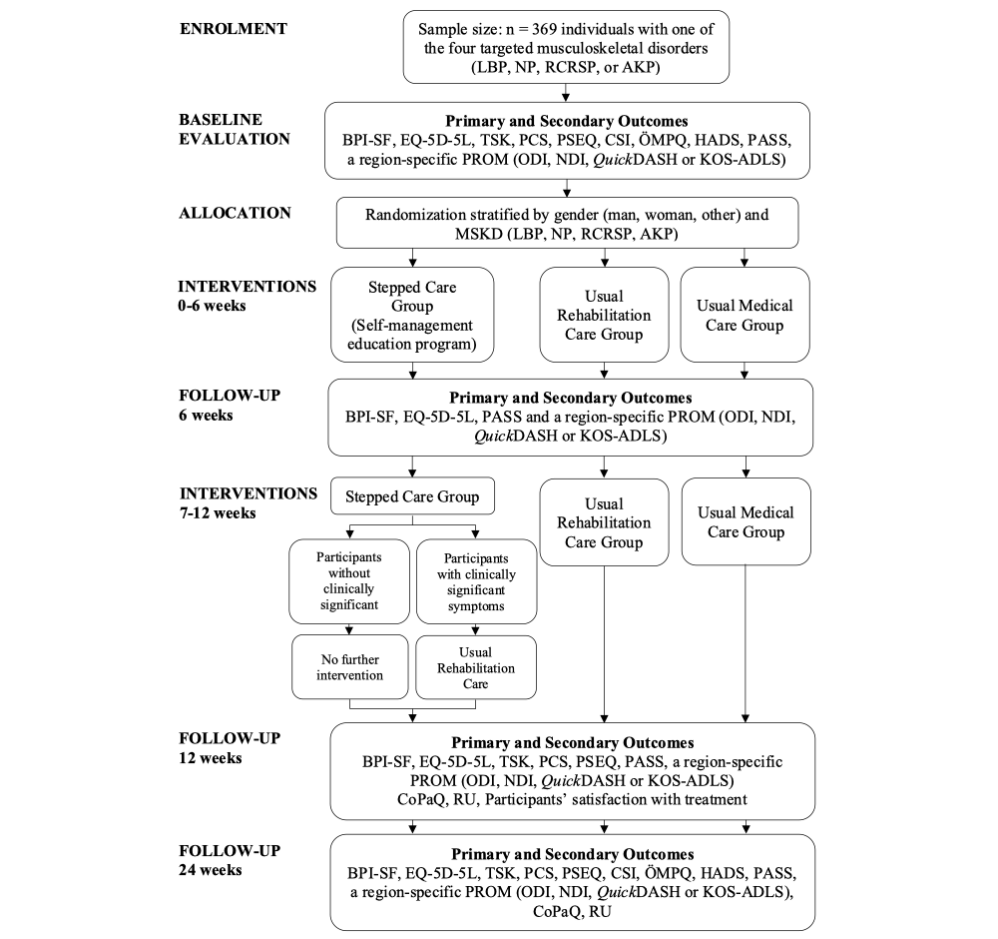
Study flow diagram.

A pragmatic, noninferiority, multiarm, parallel-group randomized controlled trial will be conducted in the Quebec City area, Canada, targeting individuals with 1of the 4 most common MSKDs encountered in primary care: low back pain, neck pain, rotator cuff–related shoulder pain, or anterior knee pain [30,31] (see Figure 1). This pragmatic trial will reflect real-world clinical practice, as participants will receive care in routine medical and rehabilitation settings [32-37]. The trial is registered at ClinicalTrials.gov (NCT06832852), and follows the multiarm extension of the Consolidated Standards of Reporting Trials (CONSORT) guidelines [38], as well as the Standard Protocol Items: Recommendations for Interventional Trials (SPIRIT) guidelines [39,40]. Interventions in each arm are described according to the Template for Intervention Description and Replication (TIDieR) checklist [41].

Participants will be recruited through a convenience sample using various channels, including social media platforms (Facebook, LinkedIn, and Instagram), electronic mailing lists of students, employees, and retirees at Université Laval, and advertisements in local newspapers. Interested individuals will be invited to contact the research team by email or phone. Initial eligibility screening will be conducted online using a web-based questionnaire through REDCap (Research Electronic Data Capture; Vanderbilt University) platform [42]. During this process, potential participants will receive information outlining the nature and objectives of the study. A follow-up phone interview with a team member will provide further eligibility screening and an opportunity to discuss the study details.

**Figure 2:**
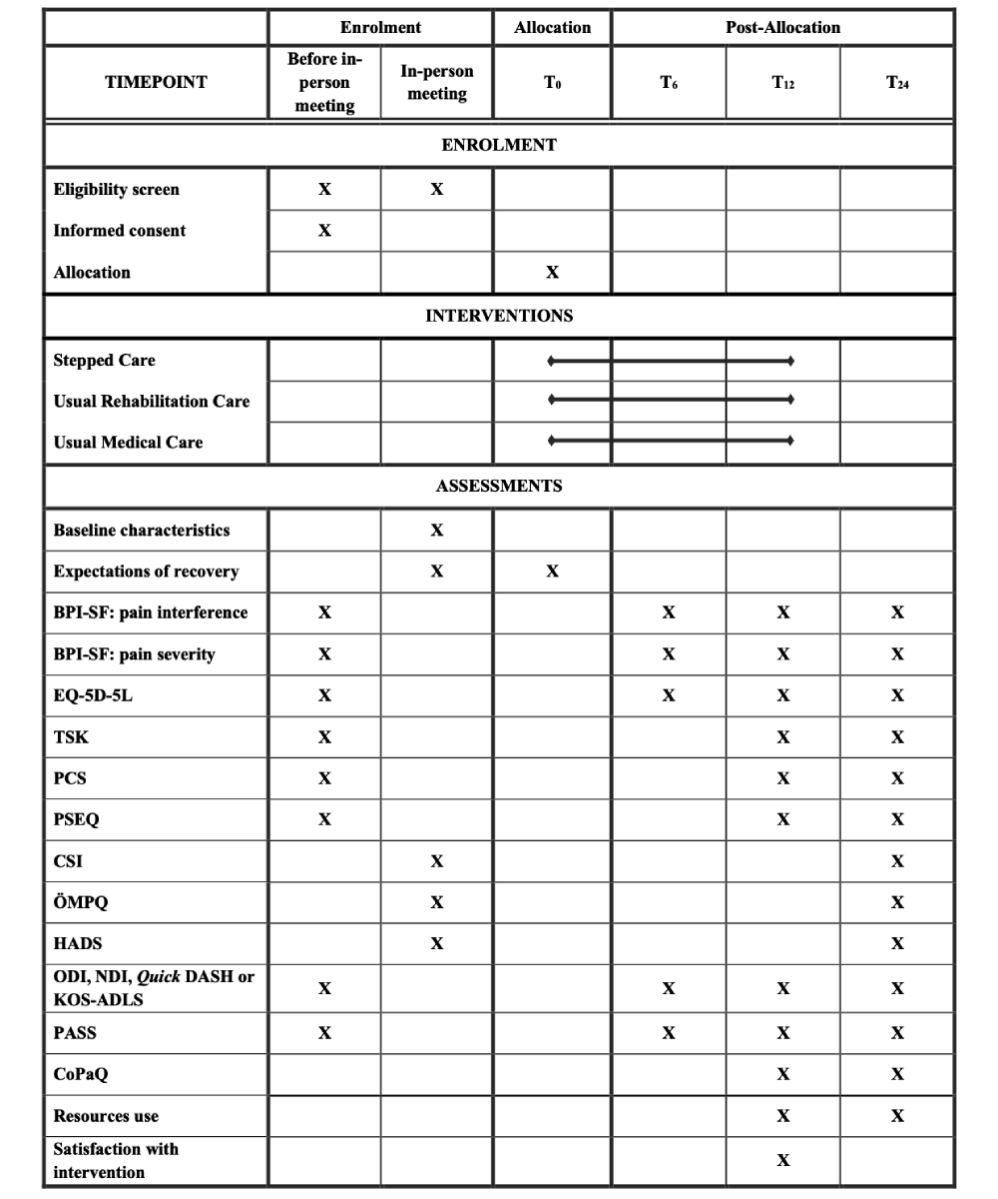
Standard Protocol Items: Recommendations for Interventional Trials (SPIRIT) diagram of enrolment, interventions, and assessments. BPI-SF: Brief Pain Inventory–Short Form; CoPaQ: Cost for Patients Questionnaire; CSI: Central Sensitization Inventory; HADS: Hospital Anxiety and Depression Scale; KOS-ADLS: Knee Outcome Survey Activities of Daily Living Scale; NDI: Neck Disability Index; ODI: modified Oswestry Disability Index; ÖMPQ: OREBRO musculoskeletal pain questionnaire; PASS: Patient Acceptable Symptom State; PCS: Pain Catastrophizing Scale; PSEQ: Pain Self-Efficacy Questionnaire; QuickDASH: shortened version of the Disabilities of the Arm, Shoulder, and Hand; TSK: Tampa Scale of Kinesiophobia.

Individuals who meet the initial criteria will be asked to provide electronic informed consent, complete the primary and some secondary outcome measures using web-based patient-reported outcome measures within REDCap (see Figure 2), and attend an in-person meeting at the Center for Interdisciplinary Research in Rehabilitation and Social Integration (Cirris), a research center affiliated with Université Laval in Quebec City. During this meeting, an experienced physiotherapist will confirm eligibility using standardized screening questionnaires and, for certain diagnoses (eg, rotator cuff–related shoulder pain and anterior knee pain), additional physical tests. All screening questionnaires used throughout the recruitment process are provided in Multimedia Appendix 1.

During the in-person meeting, enrolled participants will also complete the baseline evaluation, which includes questionnaires on sociodemographic characteristics (including sex at birth and gender identity), medical history, symptom onset context, work restrictions, recovery expectations (participants’ expectations regarding the intervention’s potential effectiveness on their pain, work capacity, and ability to engage in leisure activities) [43], and treatment preferences (eg, preferred health care provider, desired duration and frequency of consultations) [44,45]. Finally, the remaining secondary outcome measures will be evaluated on-site using web-based patient-reported outcome measures in REDCap. Participants will then be randomly assigned to one of three 12-week intervention groups: stepped care, usual rehabilitation care, or usual medical care. Recovery expectations will be reassessed immediately after participants receive an explanation of their assigned intervention.

Primary and secondary outcome measures will be reassessed online using web-based patient-reported outcome measures in REDCap at 6, 12, and 24 weeks post allocation (see Figures 1 and 2).

### Population and Sample Size

Participants must be aged between 18 and 65 years, present with 1 of the 4 targeted MSKDs (low back pain, neck pain, rotator cuff–related shoulder pain, and anterior knee pain), and have experienced pain for at least 6 weeks. Potential participants will be excluded if they: (1) are unavailable for the 12-week intervention; (2) cannot understand or read French or English; (3) have a diagnosis of rheumatoid, inflammatory or neurodegenerative diseases; (4) have signs of upper (eg, bilateral paresthesia, hyperreflexia, or spasticity) or lower (eg, decreased sensation or strength in dermatomes and myotomes, hypotonia, or hyporeflexia) motor neuron lesions; (5) have received a corticosteroid injection or any other type of injection (eg, platelet-rich plasma, prolotherapy, and hyaluronic acid) in the past 3 months for their current condition; and (6) have cognitive impairments interfering with participation (mini—mental state examination administered if doubts arise; score must be ≥24) [46,47]. Participants who previously received conservative management will be eligible if at least 6 weeks have passed since their last intervention (including prescribed condition-specific exercises or medication). Specific inclusion and exclusion criteria for each MSKDs are the following.

#### Low Back Pain

Inclusion criteria are (1) nonspecific low back pain with or without radiation to the lower limbs, (2) minimum score of 15/100 on the modified Oswestry Disability Index (ODI; 1.5 times the minimally clinically important difference [48], to ensure participants have clinically significant symptoms). Nonspecific low back pain is defined as low back pain that cannot be attributed to a specific condition. Exclusion criteria are (1) low back pain related to specific conditions (eg, vertebral fracture, infections, and neuropathic pain, with a score of ≥4 on the DN4 [Douleur Neuropathique 4, a screening questionnaire used to identify neuropathic pain] questionnaire [49]) and (2) history of spine surgery.

#### Neck Pain

Inclusion criteria are (1) nonspecific neck pain with or without radiation to the upper limbs and (2) a minimum score of 21/100 on the Neck Disability Index (1.5 times the minimally clinically important difference [50]) to ensure participants have clinically significant symptoms). Exclusion criteria are (1) neck pain related to specific conditions (eg, vertebral fracture, infections, and neuropathic pain; ≥4 on the DN4 questionnaire [49]), (2) history of spine surgery, or (3) signs of upper or lower motor neuron lesions.

#### Rotator Cuff–Related Shoulder Pain

Inclusion criteria are (1) shoulder pain attributed to rotator cuff–related shoulder pain (pain over the deltoid or upper arm region, pain associated with arm movement [painful arc of movement], and familiar pain reproduced with loading or resisted testing during external rotation of the arm) [36,51], and (2) a minimum score of 15/100 on the shortened version of the Disabilities of the Arm, Shoulder, and Hand (QuickDASH; 1.5 times the minimally clinically important difference [52], to ensure participants have clinically significant symptoms). Exclusion criteria are (1) history of shoulder surgery, dislocation, or fracture; (2) presence of severe osteoarthritis, symptomatic acromioclavicular joint pathology, or adhesive capsulitis (defined as restriction of passive glenohumeral movement of at least 30% for two or more directions); and (3) full thickness rotator cuff tear, identified by imaging or clinical tests (lag signs and gross weakness) [53,54].

#### Anterior Knee Pain

Inclusion criteria are (1) anterior knee pain during walking, running or going up or down stairs, or during at least two activities among: kneeling, squatting, and resisted knee extension [55] and (2) maximum score of 79/100 on the Knee Outcome Survey Activities of Daily Living Scale (1.5 times the minimally clinically important difference [56], to ensure participants have clinically significant symptoms). Exclusion criteria are (1) history of knee surgery or patellar dislocation, and (2) pain believed to originate either from the meniscus [57] or any knee ligament based on clinical tests.

The primary objective is to assess the equivalence between the stepped care model and the 2 usual care models. With 123 participants per group, we will have 80% power to test the noninferiority of the stepped care model compared to usual rehabilitation care and usual medical care, using a noninferiority margin of 2.0 (out of 10) points on the primary outcome measure. This margin is based on the highest suggested minimally clinically important difference for the Pain Interference subscale of the Brief Pain Inventory—Short Form (BPI-SF) [58]. The sample size calculation assumes a true mean difference of 0 between groups, and an SD of 1.8 in the usual care groups [59], a 15% dropout rate at the 24-week follow-up [20,60-62], and a 1-sided alpha level of .025.

### Randomization and Blinding

Randomization lists (1:1:1) were generated using a computer-based random number generator by an independent research assistant not involved in the project. Blocked randomization (with random block sizes of 3, 6, and 9) was used to ensure balanced group allocation. Given that randomization was stratified by gender (men, women, and other) and MSKD (low back pain, neck pain, rotator cuff-related shoulder pain, and anterior knee pain), 12 separate lists were generated. The randomization lists were integrated into the REDCap randomization module, eliminating the need for sealed envelopes. After the baseline evaluation, the research associate will enter the participant’s gender and MSKD type into REDCap, which will then generate the assigned group. The allocation will appear in a secure, concealed table within REDCap, preventing any modifications. The research associate will then inform the participant and the medical or physiotherapy clinic of the assigned group to schedule the participant’s first appointment.

Blinding of treatment providers and participants is not feasible due to the nature of the interventions. However, the statistician conducting the analyses will remain blinded to group allocation. Since all study outcomes are self-reported and collected via REDCap, an outcome assessor is not required. Participants will interact with a research associate only at baseline, before randomization. Participants will be informed that they are enrolled in 1 of 3 groups, that all groups receive high-value, individualized care, and that the number and frequency of follow-up sessions may vary. However, they will not be told whether they are assigned to a stepped care, usual rehabilitation care, or usual medical care, thereby maintaining partial blinding regarding group allocation. They will only be informed about the interventions received by participants in the other groups after completing the 24-week follow-up. To minimize potential contamination, each group will receive treatment in separate clinics: Usual medical care in medical clinics, while stepped care and usual rehabilitation care will be provided in different physiotherapy clinics. Co-interventions will be monitored at each follow-up using a questionnaire on REDCap.

### Intervention

Usual medical care will be provided by family physicians practicing in family medicine groups or university-affiliated family medicine groups, while usual rehabilitation care and stepped care will be provided by physiotherapists practicing in physiotherapy clinics across the Quebec City area. To enhance the generalizability of findings to the broader health care workforce, family physicians and physiotherapists with varying levels of experience, from early-career to experienced clinicians, will be included. Each intervention will have a maximum duration of 12 weeks. All consultation fees with the family physicians and physiotherapists related to the study will be covered by the research team.

### Stepped Care Group

During the first 6 weeks, participants in the stepped care group will engage in a web-based self-management education program, supplemented by 2 individual educational sessions with a physiotherapist. The first session (60 minutes) will begin with a clinical evaluation by the physiotherapist responsible for the educational intervention, followed by an education session covering the anatomy, function, and diagnosis of the affected area, basics of pain neuroscience, and condition-specific guidance on load management [27]. Participants will also receive strategies for pain and activity (pacing) self-management, optimal sleep and work positions, and key lifestyle factors such as stress management and nutrition [19,63]. The importance of physical activity will be emphasized, with recommendations aligned with World Health Organization guidelines (ie, 150-300 minutes of moderate-intensity activity per week), and adapted to each participant’s condition and physical capacity. Throughout the session, physiotherapists will foster open dialogue, personalizing discussions to address each participant’s needs and goals, answering questions, dispelling myths or misconceptions, providing reassurance, and discussing prognosis. An educational website, developed in collaboration with 7 patient partners, will also be introduced. It offers additional resources, including educational videos, summaries of key topics, and supplementary material for reference. Participants will be encouraged to explore these resources independently during the following weeks. The research team will have access to data on time spent on the website throughout the study.

The second educational session (30 minutes), scheduled 2 to 4 weeks later, will focus on reinforcing key concepts and providing long-term recommendations. The physiotherapist will begin by reviewing the participant’s condition and assessing their understanding of the material from the first session and the website. They will verify whether the participant has engaged with the educational website’s online content, address any remaining questions, offer personalized guidance, and provide recommendations for long-term self-management. While the second educational session will be preferably conducted in person, it may also be held via telehealth, depending on the participant’s preference. Following each session, the physiotherapist will complete a brief REDCap questionnaire documenting the topics discussed.

At the 6-week follow-up evaluation, participants in the stepped care group will be assessed using a region-specific patient-reported outcome measure to determine if their condition has resolved. Participants who no longer experience clinically important symptoms at the 6-week follow-up will be classified as “resolved” and will not receive further intervention. Those with scores exceeding the minimal clinically important difference thresholds on their region-specific patient-reported outcome measure (low back pain >10 on the ODI; neck pain >14 on the Neck Disability Index [NDI]; rotator cuff-related shoulder pain >10 on the QuickDASH; and anterior knee pain <86 on the Knee Outcome Survey Activities of Daily Living Scale [KOS-ADLS]) will be considered “unresolved” and will receive follow-up rehabilitation interventions. This dichotomization approach between “resolved” and “unresolved” MSKDs was chosen because, while an improvement greater than the minimal clinically important difference would be indicative of clinically significant improvement, participants may still experience substantial symptoms and limitations if their total score remains above the minimal clinically important difference threshold [64]. Participants classified as “unresolved” will undergo a rehabilitation intervention supervised by a physiotherapist over the following 6 weeks. This intervention will mirror the usual rehabilitation care group’s approach but will be condensed into a maximum of 5 sessions within a 6-week period. To prevent bias in patient-reported outcome measures responses, participants will not be informed during the first 6 weeks that they may be eligible for additional physiotherapy sessions if their symptoms remain clinically significant at the 6-week follow-up. Similarly, participants classified as “resolved” will not be informed at the 6-week mark that additional treatments could have been offered if their symptoms had persisted after the educational intervention. This information will only be disclosed at the end of the study, at the 24-week follow-up.

### Usual Rehabilitation Care Group

Participants will engage in a pragmatic 12-week rehabilitation intervention led by a physiotherapist, consisting of up to 10 individualized sessions, each lasting 30 minutes (except for the initial 60-minute session), along with an individualized home exercise program. During the first session, the physiotherapist will conduct an initial clinical evaluation. Given the pragmatic nature of the intervention, its structure and modalities will remain flexible, shaped collaboratively by the physiotherapist and the participant. This approach will take into account the participant’s goals and preferences, as well as the observed disabilities and the severity of the condition. Exercises may be used to improve strength, endurance, flexibility, the ability to sustain mechanical load, and dynamic control, as needed. In addition, participants may receive educational information related to their painful area, along with advice on pain and load management, activity modification, and any other relevant topics as determined by the treating physiotherapist. Manual therapy techniques (eg, massage, mobilization, or manipulation) and other passive interventions (eg, electrotherapy, heat, ice, dry needling, or any type of taping) may be used during sessions based on pragmatic clinical decision-making [65]. At the end of each supervised session, participants may receive individualized exercises, with progression, to perform at home between sessions.

If participants become symptom-free before completing the 10 sessions, or if the participant and physiotherapist determine that no further sessions are needed, treatment will be discontinued. Participants will then receive advice, recommendations, and home exercises as appropriate. If needed, they will have the option to resume the intervention within the 12-week period. Participants will not be restrained from using over-the-counter medications for pain relief if deemed helpful. If pain persists, the physiotherapist may refer participants to a family physician for further medical evaluation and management, which may include diagnostic tests (clinical, laboratory, or imaging), pharmacological pain management, and corticosteroid injections. Participants may also be referred to other health care providers (eg, psychologists, occupational therapists, and kinesiologists). However, these referrals will not be managed by the research team, and the associated costs will be the participant's responsibility. After each supervised session, the physiotherapist will complete a short REDCap questionnaire detailing the intervention modalities used, exercises prescribed, any referrals made, and the follow-up plan.

### Usual Medical Care Group

Participants will take part in a pragmatic 12-week medical intervention led by a family physician, consisting of up to 3 consultations during this period. The initial in-person meeting at the medical clinic will involve a comprehensive evaluation by the family physician, which may include diagnostic clinical tests, laboratory tests, and medical imaging if necessary. Based on this assessment, the family physician and participant will collaboratively determine the most appropriate management approach. Interventions may range from reassurance and patient education on pain management strategies to pharmacological treatments and corticosteroid injections. If needed, the family physician may refer participants to a physiotherapist, medical specialist, or other health care providers (eg, psychologists, occupational therapists, kinesiologists, acupuncturists, or chiropractors). However, these referrals will not be managed by the research team, and the associated costs will be the participant's responsibility.

During the 2 potential follow-up meetings, the participant’s condition will be reassessed, and the intervention will be adjusted accordingly. While these meetings are preferably conducted in person, they may also be held by phone or via telehealth, depending on the participant’s preference. If a participant becomes symptom-free before completing the 3 sessions, or if the participant and family physician decide that no further sessions are required, treatment will be discontinued, and appropriate advice and recommendations will be provided. However, they will have the option to resume the intervention within the 12-week period if needed. Given the pragmatic nature of the intervention, its structure remains flexible, allowing for adaptation based on the participant’s goals and preferences and clinical presentation. Following each session, the family physician will complete a brief REDCap questionnaire documenting the management approach used (eg, education, medication, and injection), any imaging or laboratory tests ordered, any referrals made, and the follow-up plan.

### Training of Treatment Providers

Given the pragmatic nature of the interventions in the usual medical care and usual rehabilitation care groups, the physiotherapists and family physicians in these groups will not receive specific training related to the intervention. However, they will attend a 60-minute meeting outlining the study objectives and relevant procedures (eg, maximum number of consultations, the brief questionnaire to be completed in REDCap, and the expectation that they provide care according to their usual practices).

Physiotherapists delivering the educational intervention were selected based on their expertise in a clinic specializing in educational approaches for MSKDs. They will participate in a 2-hour in-person training session led by 3 members of the research team. This training will emphasize the educational components to be delivered and the importance of establishing a strong therapeutic alliance with participants. The study’s educational website will also be presented and explained during this session. To ensure consistency, follow-up meetings will be held twice a year to discuss strategies for maintaining standardization and to address any challenges that arise.

It should be noted that physiotherapists and family physicians providing usual medical care and usual rehabilitation care will not have access to the educational website, nor will it be presented to them.

### Primary and Secondary Outcome Measures

#### Overview

The study outcomes will be assessed online using web-based patient-reported outcome measures on REDCap [42]. Patient-reported outcome measures integrated within REDCap are designed to prevent incomplete submissions, ensuring that all questionnaires are fully completed. The primary outcome will be functional limitations at 24 weeks, as measured with the Pain Interference subscale of the BPI-SF. Secondary outcomes include pain severity as measured with Pain Severity subscale of the BPI-SF, health-related quality of life (HRQoL) as measured with the EQ-5D-5L, pain-related fear as measured with Tampa Scale for Kinesiophobia (TSK), pain catastrophizing as measured with Pain Catastrophizing Scale (PCS), pain self-efficacy as measured with Pain Self-Efficacy Questionnaire (PSEQ), symptoms related to central sensitization using the Central Sensitization Inventory (CSI), risk of long-term disability and poor recovery as measured with the short version of the OREBRO musculoskeletal pain questionnaire (ÖMPQ-sf), and anxiety and depressive symptoms as measured with Hospital Anxiety and Depression Scale (HADS). Furthermore, region-specific symptoms and functional limitations will be assessed at all time points using the modified ODI for participants with low back pain, the NDI for those with neck pain, the QuickDASH for participants with rotator cuff–related shoulder pain, and the KOS-ADLS for those with anterior knee pain. Finally, participants’ satisfaction with the treatment will be assessed at the end of the intervention period (week 6 for the stepped care group, and week 12 for the “unresolved” participants in the stepped care, usual rehabilitation, and usual medical care groups). Validated French or English versions of all questionnaires are available. The psychometric properties of all patient-reported outcome measures have been established in populations with MSKDs [66-70]. Given the number of patient-reported outcome measures assessed at baseline, their completion will be divided into 2 sequences. The first sequence (BPI-SF, EQ-5D-5L, TSK, PCS, PSEQ, and region-specific patient-reported outcome measure) will be completed before the in-person meeting, while the second sequence (CSI-9, ÖMPQ-sf, and HADS) will be completed during the in-person meeting. Although most outcomes will be assessed at all measurement time points, some secondary outcomes will only be reassessed at 12 or 24 weeks to minimize the burden on participants. Figure 2 provides an overview of when the patient-reported outcome measures will be assessed at the different measurement time points.

#### Primary Outcome: Functional limitations

The BPI-SF is an 11-item questionnaire comprising two subscales: the 4-item Pain Severity subscale and the 7-item Pain Interference subscale. It has been shown to be valid, reliable, and responsive for MSKDs [71-74]. The Pain Interference subscale, measured at the 24-week follow-up, will be the primary outcome measure, assessing the extent to which pain disrupts daily functioning [73,74]. Its minimally clinically important difference ranges from 1.3 to 2.0 out of 10 points [58].

#### Secondary Outcomes: Generic Patient–Reported Outcome Measures

Secondary outcomes comprised pain severity, HRQoL, pain-related fear, pain catastrophizing, pain self-efficacy, symptoms related to central sensitization, risk of long-term disability, and anxiety and depressive symptoms. Pain severity: The Pain Severity subscale of the BPI-SF consists of 4 items that assess pain intensity [75]. HRQoL: The EQ-5D-5L is a 5-item generic HRQoL questionnaire evaluating five dimensions: mobility, self-care, usual activities, pain and discomfort, and anxiety and depression. These dimensions define 55=3125 possible health states, which can be converted into a Canadian weighted index score [76]. The weighted index scores at each time point will be used to calculate the quality-adjusted life years for each participant. Quality-adjusted life years will be the primary outcome measure for the cost-effectiveness analysis and will be used to estimate the incremental cost-effectiveness ratio (ICER) [66]. Pain-related fear: The TSK is a questionnaire assessing beliefs and behaviors related to pain, with a focus on the perception that pain is damaging [77]. The 13-item version will be used. Pain catastrophizing: The PCS is a questionnaire designed to identify catastrophic thoughts about pain [78]. The 13-item version will be used. Pain self-efficacy: The PSEQ measures a patient’s confidence in performing daily activities despite pain [79]. The 10-item version will be used. Symptoms related to central sensitization: The CSI evaluates the frequency of common symptoms associated with central sensitization [80,81]. The 9-item version will be used. Risk of long-term disability: The ÖMPQ-sf is a questionnaire that assesses psychosocial and pain-related factors to predict the risk of long-term disability and delayed return to work in individuals with MSKDs [82]. The 10-item version will be used. Anxiety and depressive symptoms: The HADS is a 14-item questionnaire used to screen the presence of anxiety and depression [70].

#### Secondary Outcomes: Region-Specific Symptoms and Functional Limitations Patient-Reported Outcome Measures

Participants will only complete the region-specific patient-reported outcome measure relevant to their condition. Low back pain: the modified ODI (minimal detectable change=10 points and minimally clinically important difference=10 points) is a 10-item questionnaire assessing the impact of low back pain on activities of daily living [48,83-86]. Neck pain: NDI (minimal detectable change=10 points and minimally clinically important difference=14 points [50,87]) is a 10-item questionnaire measuring self-reported disability related to neck pain [50]. Rotator cuff-related shoulder pain: QuickDASH (minimal detectable change=11 points and minimally clinically important difference=10 points) is an 11-item questionnaire, validated in individuals with rotator cuff–related shoulder pain [88], that assesses difficulty in performing daily activities and the severity of the upper limb symptoms [89]. Anterior knee pain: KOS-ADLS (minimal detectable change=8.3 points and minimally clinically important difference=14 points) is a 14-item knee-specific questionnaire that evaluates symptoms and functional limitations [90].

#### Secondary Outcomes: Participants’ Satisfaction

Participants’ satisfaction with their condition will be assessed at each evaluation time point using the Patient Acceptable Symptom State [64,91]. This measure asks patients whether they consider their current state acceptable and requires them to rate their satisfaction on a 0-100 numeric scale [73]. At the end of the intervention, participants will also evaluate their satisfaction with various aspects of the treatment received. They will rate their overall satisfaction (“not satisfied,” “satisfied,” and “very much satisfied”), the adequacy of treatment duration (“too short,” “long enough,” and “too long”), treatment frequency, and the time spent with the health care providers during sessions (“not enough,” “just right,” and “too much”) using a 3-item Likert scale [73,75,92].

#### Secondary Outcomes: Health Care Resource Use and Health Costs

At the 12-week (covering weeks 0 to 12) and 24-week (covering weeks 13 to 24) follow-up assessments, participants will complete 2 questionnaires: one assessing resource use from the perspective of both public health services and private insurers, and another capturing out-of-pocket costs incurred by participants. For the latter, the Cost for Patients Questionnaire (CoPaQ) will be used, a validated tool that measures patient-related costs, including those incurred by informal caregivers [93,94]. The CoPaQ covers expenses such as copayments, transportation to treatment sessions, childcare, time off work, and lost income. The resource use questionnaire will collect data on health care use, including visits to family physicians, physiotherapists, and other health care providers, as well as medical imaging, laboratory tests, and indirect costs. It will also collect information on each participant’s private health insurance and the extent of coverage for each resource used, where applicable. The average cost of consultations with each type of provider will be determined based on reimbursement rates from both the public and private payers.

### Data Integrity and Analysis

All collected data will be securely stored on a server hosted at Université Laval, with access restricted to the research team. Data will be retained for 7 years after the study’s completion to support planned publications, after which it will be permanently deleted. Given the low-risk nature of this trial, a Data Monitoring Committee is not required, and no interim analysis will be conducted.

### Statistical Analysis Plan

Descriptive statistics will be used to summarize participants’ characteristics and outcome measures at the different measurement times. Participants who withdraw from the study and the reasons for withdrawal will be analyzed. Any harm or unintended effects will be recorded by both the treating family physician and physiotherapist at the beginning of each consultation, as well as by the participants during the follow-up evaluations on REDCap. If a participant experiences an adverse event, the principal investigator will report it to the Ethics Committee. However, since the interventions included in this trial are commonly used in clinical practice and are generally well tolerated, no major adverse events directly related to the intervention are expected, except perhaps a temporary increase in pain.

For each of the primary and secondary patient-reported outcome measures, repeated measures analyses will be performed using linear mixed models with a random effect by participant to account for the correlation present in repeated measures within individuals. The model allows assessment of the group and time effects, as well as the group-by-time interaction. An intention-to-treat analysis will be used. Gender and type of MSKD will be used to test the interaction between the group effect and the strata in order to test whether the comparison across interventions depends on gender or type of MSKD. Residual analysis will be performed to verify that the assumptions of normality and homoscedasticity are met. If not, the model will be extended to a generalized linear mixed-model framework. Effect sizes will be presented as the standardized differences between treatment groups in the mean weekly improvements over 12 and 24 weeks. Satisfaction will be compared using chi-squared tests, Mann-Whitney tests, or independent *t* tests.

For economic analyses, the total cost of an episode of care will be calculated from the perspectives of the public health service, the participants, and private insurers. ICERs will then be computed from 3 perspectives: the public health service, the participant, and society. The ICERs will be measured as the difference between the mean costs divided by the difference in effectiveness at 24 weeks between the stepped care model and each of the usual care models [95].

### Mitigation Plan

As with any clinical trial, maintaining adherence to the intervention and follow-ups may be challenging for some participants. To encourage compliance, several strategies will be implemented. Medical and rehabilitation interventions will be held in clinics offering both daytime and evening appointment options. Follow-up assessments will be conducted online to minimize travel requirements. Automated reminders will be sent via email as needed, and research associates will be available to provide assistance via email or phone. In addition, participants will receive CAD $40 (approximately US $28.50 as of October 2025) at the baseline evaluation to cover travel expenses and another CAD $40 (approximately US $28.50 as of October 2025) at the end of the project to compensate for time spent completing follow-up questionnaires.

### Ethical Considerations

Ethics approval has been granted by the Comité d’éthique de la recherche sectoriel en réadaptation et intégration sociale du CIUSSS de la Capitale-Nationale (Rehabilitation and Social Integration section; #2024-2982). All participants will receive a comprehensive information and consent form outlining the nature and objectives of the study.

Participation is entirely voluntary. Participants may decline to take part or withdraw from the study at any time, without providing a reason, by informing the principal investigator or a member of the project staff. They may also request that any data collected from them be withdrawn.

Electronic informed consent will be required before enrollment. Participants’ confidentiality will be maintained by the research team throughout the project in accordance with current regulations. Participants’ identities will be coded, and all data will be stored in secure folders within the research team’s offices or in password-protected files. It will not be possible to identify participants in any publications.

### Patient and Public Involvement

The website used during the first six weeks of the stepped care intervention was co-created with seven patient-partners living with chronic MSKDs (mean age 49.4 y, SD 14.7 y; 71% women). The website underwent iterative development until it received approval from the patient-partners. They also provided feedback on the selected set of patient-reported outcome measures to ensure that the completion burden was acceptable and that all important constructs were adequately assessed. Additionally, a patient partner contributed to the design of the study protocol to ensure its relevance and alignment with patient needs.

## Results

The Edu-First trial is funded by a project grant from the Canadian Institutes of Health Research (grant #495615), with funding spanning from October 2023 to September 2028.

Recruitment began on January 31, 2025, with the first participants enrolled in February 2025. As of September 2025, a total of 65 participants have been enrolled. Recruitment is expected to continue for up to 3 years, aiming for approximately 10 new participants per month, and is anticipated to be completed by Winter 2028. Data analysis and manuscript preparation are projected to take up to 6 months, with the first manuscripts anticipated for submission by Fall 2028.

## Discussion

### Principal Findings

We anticipate that the stepped care model will be noninferior to usual medical care and usual rehabilitation care in terms of treatment effectiveness. In addition, we expect this model to reduce health care costs by limiting the use of expensive resources, such as consultations with health care providers and medical investigations. As health care costs for MSKDs continue to rise, implementing a stepped care model—where patient education and self-management advice are prioritized initially, and usual care is reserved for those whose symptoms persist—could improve access to care without compromising quality. This approach also empowers patients to take an active role in managing their condition, overall health, and well-being from the outset.

MSKDs are among the most common reasons for primary care consultations, with up to 20% of adults seeking care for these conditions annually [8], and approximately 30%-40% of individuals with MSKDs reporting contacting their family physician for these complaints [30]. Despite their high prevalence, MSKDs remain difficult to treat effectively beyond the acute phase [96-98]. Given the significant impact of MSKDs on individuals’ activities and participation, as well as their contribution to societal burdens such as work loss, disability pensions, early retirement, and increased demand for social support [3,4], it is crucial to explore innovative care models to address these challenges.

The findings from this study could inform systemic changes in care delivery, improving access to treatment while reducing the average cost per care episode. Ultimately, this study aims to provide robust evidence supporting more efficient, patient-centered care models for a prevalent and costly set of conditions.

### Strengths and Limitations

This study is designed as a pragmatic randomized controlled trial, reflecting real-world clinical practice and enhancing the generalizability of its findings. Key strengths include the direct comparison of the stepped care model with 2 of the most widely used care models, the inclusion of cost-effectiveness analyses, and the focus on patient-centered outcomes. Potential limitations include the reliance on patient-reported data for some outcomes, which may introduce reporting bias, and the heterogeneity of MSKD presentations, which could influence treatment response. In addition, given the pragmatic nature of the trial, variability in usual care practices may impact the consistency of comparator interventions.

### Future Directions

If the stepped care model proves to be noninferior in effectiveness and superior in cost-effectiveness, the results could guide systemic improvements in the delivery of MSKD care. Wider implementation of this model may improve access to health care services, particularly in primary care settings, while reducing the economic burden on both patients and the health care system. Future research could explore long-term outcomes, patient adherence to self-management strategies, and the applicability of this model to other musculoskeletal conditions or health care systems.

### Dissemination Plan

At the conclusion of the project, targeted materials will be developed for patients, clinicians, managers, and decision-makers. These will include infographics, podcasts, webinars, presentations, and videos, which will be shared through a variety of clinical and community platforms, including the websites and social media channels of professional associations and key partners. Academic dissemination will include peer-reviewed manuscripts and conference presentations to ensure broad visibility within the scientific community.

## Appendix

Multimedia Appendix 1 Recruitment process.

Multimedia Appendix 2 Peer-review report by the Project. Grant / Subvention Projet - Clinical Investigation - B 2 / Investigation clinique B 2 - Canadian Institutes of Health Research (CIHR) / Instituts de recherche en santé du Canada (IRSC).

## Abbreviations

BPI-SF: Brief Pain Inventory – Short Form
CoPaQ: Cost for Patients Questionnaire
CONSORT: Consolidated Standards of Reporting Trials
CSI: Central Sensitization Inventory
HADS: Hospital Anxiety and Depression Scale
HRQoL: health-related quality of life
ICER: incremental cost-effectiveness ratio
KOS-ADLS: Knee Outcome Survey Activities of Daily Living Scale
MSKD: musculoskeletal disorder
NDI: Neck Disability Index
ODI: modified Oswestry Disability Index
ÖMPQ: OREBRO musculoskeletal pain questionnaire
PCS: Pain Catastrophizing Scale
PSEQ: Pain Self-Efficacy Questionnaire
QuickDASH: shortened version of the Disabilities of the Arm, Shoulder, and Hand
REDCap: Research Electronic Data Capture
SPIRIT: Standard Protocol Items: Recommendations for Interventional Trials
TIDieR: Template for Intervention Description and Replication
TSK: Tampa Scale of Kinesiophobia
