# Supplementary material for: Effectiveness and Cost-Effectiveness of a Stepped Model of Care for Musculoskeletal Disorders: Protocol for a Multiarm Randomized Controlled Trial (Edu-First Trial)
Source: JMIR Res Protoc. 2025 Nov 19;14:e77574. doi: 10.2196/77574 (PMC12676218; doi:10.2196/77574)
Supplement: Multimedia Appendix 1 [file resprot_v14i1e77574_app1.docx]

**Supplementary File 1**

**Step 1 - Eligibility questionnaire on REDCap**

**1. Age**
How old are you? ____ years

**2. Language**
Do you understand French or English, both in writing and when spoken?
☐ Yes  ☐ No

**3. Pain location**
Where is your pain located?
If you have pain in more than one of the areas listed, please select the one that is most disabling.
☐ Low back
☐ Neck
☐ Shoulder
☐ Knee
☐ Other (specify): ____________________

**4. Diagnosis**
Have you received a diagnosis for this problem?
☐ Yes  ☐ No

If yes, please specify the diagnosis (name only, if known): ____________________

**5. Other conditions**

Do you have any other significant musculoskeletal pain or disability?

☐ Yes  ☐ No
If yes, indicate the body part concerned: ____________________

Have you ever been diagnosed with a rheumatic disease (e.g., rheumatoid arthritis), an inflammatory or neurodegenerative disease, or fibromyalgia?
☐ Yes  ☐ No

If yes, please specify: ____________________

**6. Duration of pain**
How long have you been experiencing this pain?
(If the problem is episodic or intermittent, please indicate the duration of the current episode)
☐ Less than 6 weeks
☐ 6 weeks to 3 months
☐ 3 months to 1 year
☐ 1 year to 5 years
☐ More than 5 years

**7. Commitment**
Are you confident in your ability to commit to follow-up with a healthcare professional for up to 12 weeks, including travel for in-person visits?
☐ Yes  ☐ No

**8. Injections**
Have you received an injection (e.g., cortisone) in the last 3 months?
☐ Yes  ☐ No

If yes, please specify (as best as you can):

- Body part: ____________________
- Date (exact or approximate): ____________________
- Medication received (if known): ____________________

**9. Treatments**
In the last 3 months, have you received treatment for your pain (other than injections)?
This may include consulting a healthcare professional, taking prescribed medication, or doing prescribed exercises specific to your condition.
☐ Yes  ☐ No

If yes, please specify (as best as you can):

- Treatment(s) received and/or healthcare professionals consulted: ___________________
- Number of appointments or sessions: ____________________
- Approximate dates or duration: ____________________

______________________________________________________________________________

**Follow-up Questions Based on Pain Location**

**Note:** The following questions will be added depending on the pain location indicated in Question 3.

**For those who answered “Low back” or “Neck”**

**A. Pain characteristics (First 7 questions of the DN4)**

1. Does the pain have one or more of the following characteristics?

- Burning ☐ Yes ☐ No
- Painful cold ☐ Yes ☐ No
- Electric shocks ☐ Yes ☐ No

1. Is the pain associated with one or more of the following symptoms in the same area?

- Tingling  ☐ Yes ☐ No
- Pins and needles ☐ Yes ☐ No
- Numbness ☐ Yes ☐ No
- Itching ☐ Yes ☐ No

**B. Spinal history**
Have you ever had a spinal fracture or another specific spinal disorder (injury, disease, etc.)?
☐ Yes ☐ No

If yes, please provide as much detail as possible:

- Type of injury or disease: ____________________
- Area of the spine affected: ____________________
- When it occurred: ____________________

**C. Spinal surgery**
Have you ever had spinal surgery?
☐ Yes ☐ No

If yes, please provide as much detail as possible:

- Type of surgery: ____________________
- Area of the spine: ____________________
- Year of surgery: ____________________

**For those who answered “Shoulder”**

**A. Shoulder history**
Have you ever had:

- Shoulder surgery ☐ Yes ☐ No
- Shoulder dislocation ☐ Yes ☐ No
- Shoulder fracture ☐ Yes ☐ No
- Shoulder capsulitis ☐ Yes ☐ No
- Another type of shoulder injury ☐ Yes ☐ No

**For those who answered “Yes” to Shoulder surgery**

- Type of surgery: ____________________
- Shoulder (right/left): ____________________
- Year of surgery: ____________________

**For those who answered “Yes” to Another type of shoulder injury**

- Type of injury: ____________________
- Shoulder (right/left): ____________________
- Time or period: ____________________
- Number of episodes (if applicable): ____________________

**For those who answered “Knee”**

**A. Knee pain during activities**
Do you experience pain in your knee(s) during the following activities?

- Walking ☐ Yes ☐ No
- Running ☐ Yes ☐ No
- Stairs (up or down) ☐ Yes ☐ No
- Kneeling ☐ Yes ☐ No
- Squatting ☐ Yes ☐ No

**B. Knee history**
Have you ever had:

- Knee surgery  ☐ Yes ☐ No
- Patella (kneecap) dislocation ☐ Yes ☐ No
- Another traumatic knee injury ☐ Yes ☐ No

**For those who answered “Yes” to Knee surgery**

- Type of surgery: ____________________
- Knee (right/left): ____________________
- Year of surgery: ____________________

**For those who answered “Yes” to Another traumatic knee injury**

- Type of injury: ____________________
- Knee (right/left): ____________________
- Time or period: ____________________
- Number of episodes (if applicable): ____________________

**Region-specific symptoms and functional limitations patient-reported outcome measures** After completing the REDCap eligibility questionnaire, participants will be asked to complete a region-specific symptoms and functional limitations questionnaire specific to their pain location:

- **For those who answered “Low back” at Question 3:** modified Oswestry Disability Index (ODI)
- **For those who answered “Neck” at Question 3:** Neck Disability Index (NDI)
- **For those who answered “Shoulder” at Question 3:** QuickDASH
- **For those who answered “Knee” at Question 3:** Knee Outcome Survey – Activities of Daily Living Scale (KOS-ADLS)

**Step 2 - Phone interview**

After reviewing the responses from Step 1, potential participants who appear to meet the inclusion criteria will be contacted by phone.

**During the call, the following topics will be covered:**

- Review of all responses in the eligibility questionnaire
- Additional questions to confirm that the participant meets the study diagnosis, including:
  - History of symptom onset
  - Detailed localization and characteristics of pain
  - Range of motion limitations, particularly for shoulder and knee
  - History of interventions received, if applicable

**If the participant continues to meet all inclusion criteria:**

- Provide an explanation of the study procedures by summarizing the consent form
- Answer participant’s questions
- Schedule the initial assessment

**Step 3 – Confirmation of eligibility during an in-person meeting at the at the Center for Interdisciplinary Research in Rehabilitation and Social Integration (Cirris)**

During this visit, the following will take place:

1. **Review of Step 2 discussion**
   - The information collected and discussed during the phone interview (Step 2) will be reviewed for accuracy and completeness.
2. **Cognitive screening**
   - The Mini-Mental State Examination will be administered if there are doubts about cognitive impairments that could interfere with participation.
3. **Diagnosis-specific assessments**

**A. Non-specific low back pain and neck pain**

- Complete the last three questions of the DN4 questionnaire:
  1. Is the pain located in an area where physical examination may reveal one or more of the following characteristics?
- Hypoesthesia to touch ☐ Yes ☐ No
- Hypoesthesia to pinprick ☐ Yes ☐ No
  1. In the painful area, can the pain be caused or increased by:
- Brushing ☐ Yes ☐ No

These responses will be combined with the first seven DN4 questions assessed during Step 1 to calculate the total DN4 score.

- **Neurological examination** to exclude:
  1. Upper motor neuron lesions (e.g., bilateral paresis, hyperreflexia, spasticity)
  2. Lower motor neuron lesions (e.g., decreased sensation or strength in dermatomes or myotomes, hypotonia, or hyporeflexia)

**B. Rotator cuff-related shoulder pain**

- Assessment of **active range of motion** to confirm pain associated with arm movements, particularly during **arm elevation** (painful arc of movement)
- Reproduction of familiar pain with **loading or resistance testing** during humeral abduction and external rotation
- Assessment of **passive ROM** to exclude capsulitis
  - Participants presenting a mobility restriction greater than 30% in at least two movements will be excluded

**C. Anterior knee pain**

- Assessment of **passive ROM**
- **Thessaly test** to rule out meniscal injury
- **Ligament stress tests** performed as needed based on medical history
- Assessment or observation of pain reproduction during the following activities or movements:
  - Walking
  - Running
  - Going up and down stairs
  - Kneeling
  - Squatting
  - Resisted isometric knee extension
