# Supplementary material for: Effectiveness and Cost-Effectiveness of a Stepped Model of Care for Musculoskeletal Disorders: Protocol for a Multiarm Randomized Controlled Trial (Edu-First Trial)
Source: JMIR Res Protoc. 2025 Nov 19;14:e77574. doi: 10.2196/77574 (PMC12676218; doi:10.2196/77574)
Supplement: Multimedia Appendix 2 [file resprot_v14i1e77574_app2.pdf]

**Review Type / Type d'évaluation:**	Reviewer 1 / Évaluateur 1
**Name of Applicant / Nom du chercheur:**	Roy, Jean-Sébastien
**Application No. / Numéro de demande:**	485725
**Agency / Agence:**	CIHR/IRSC
**Competition / Concours:**	Project Grant/Subvention Projet
**Committee / Comité:**	Clinical Investigation - B 2/Investigation clinique - B 2
**Title / Titre:**	Is a stepped model of care cost-effective compared to usual care for chronic musculoskeletal disorders? A randomized controlled trial

Adjudication Criteria/Critères de sélection

Initial Score/Cote Initiale: 4.4

Top/Bottom Selection/Groupe supérieur/inférieur

- (●) **Top/Groupe supérieur**
- ( ) **Bottom/Groupe inférieur**

**Review Type / Type d'évaluation:**	Reviewer 1 / Évaluateur 1
**Name of Applicant / Nom du chercheur:**	Roy, Jean-Sébastien
**Application No. / Numéro de demande:**	485725
**Agency / Agence:**	CIHR/IRSC
**Competition / Concours:**	Project Grant/Subvention Projet
**Committee / Comité:**	Clinical Investigation - B 2/Investigation clinique - B 2
**Title / Titre:**	Is a stepped model of care cost-effective compared to usual care for chronic musculoskeletal disorders? A randomized controlled trial

Summary of Application/Résumé de la demande:

This is a 5-year proposal for a RCT to establish the effectiveness of a new model of care for chronic MSKDs by comparing a stepped care model to the two most widely used models of care: usual medical care and usual rehabilitation care. The hypothesis is that a stepped care model will be at least as effective as usual medical and rehabilitation care in terms of patient-related outcomes (e.g., functional limitations, pain) but will lead to lower healthcare costs.
363 adults between 18 and 65 years of age and presenting one of the four targeted MSKDs (LBP, neck pain, patellofemoral pain syndrome, rotator cuff related shoulder pain,) and have had pain for at least 3 months will be randomized to one of the intervention groups: 1) stepped care, 2) usual medical care (pharmacological pain management), 3) usual rehabilitation care (exercises, education).

Timepoints include evaluations at baseline, and after 6, 12 and 24 weeks. At each evaluation, the primary (functional limitations) at 24 weeks, and secondary outcomes (e.g. pain severity, health-related quality of life, pain-related fear) will be assessed.

Participants in the stepped care model will take part in two education sessions during the first 6 weeks. After 6 weeks those still experiencing clinically important symptoms will receive follow-up
interventions, while those not experiencing clinically important symptoms will be considered
recovered and will have no further intervention.

The team includes ECR, mid and senior researchers with expertise in medicine, rehabilitation, education, organization of health services, biostatistics and health economics from three centers (U Laval, U Montreal, CHUM). The co-applicants have experience leading RCTs in the fields of rehab, cost-effectiveness and education, and the co-Is have used KT to produce materials on pain management geared at different end users.

**Review Type / Type d'évaluation:**	Reviewer 1 / Évaluateur 1
**Name of Applicant / Nom du chercheur:**	Roy, Jean-Sébastien
**Application No. / Numéro de demande:**	485725
**Agency / Agence:**	CIHR/IRSC
**Competition / Concours:**	Project Grant/Subvention Projet
**Committee / Comité:**	Clinical Investigation - B 2/Investigation clinique - B 2
**Title / Titre:**	Is a stepped model of care cost-effective compared to usual care for chronic musculoskeletal disorders? A randomized controlled trial

Strengths and Weaknesses/Forces et faiblesses:

STRENGTHS:

-Inclusion/exclusion criteria clearly described overall as well as for each of the four MSK pain-related conditions

-involvement of a patient partner for her participation in monthly research team meetings and for taking part in dissemination activities (e.g., co-presentations)\

-Clear flow of the study, and description of the 3 intervention arms (Appendix)

-Questionnaires include targeted outcomes and aims and provided in both English and French (Appendix)

-Strong KT plan geared at various end users with LOS for dissemination of findings across relevant national and provincial networks (rehab, health care, pain, PT)

-the self-management education intervention components has been published by this group and shown to be effective in individuals with chronic MSK disorders

-potential outcomes for each intervention are provided with a clear description of the next steps- should a participant still experience pain after the education arm (if randomized to this arm), they will be included in the usual rehab group; If participants in the usual rehab care group are symptom free before the end of the 10 sessions, treatment will be ceased, and home exercises will be given (with the possibility to resume the intervention if there is a recurrence of pain within the 12-week intervention period).

-randomization: 8 lists will be provided (one for each sex for each of the 4 pain conditions) and randomization will be conducted with the Random Allocation software. RAs will be blinded

-sample size calculation appropriate and done for the experimental treatment, yielding a sample requiring 123 participants x 3 arms = 369 total (includes loss to follow-up rate of 15%)

-statistical analysis plan clear and will be stratified by sex and condition-based pain groups to verify whether the comparison across interventions depends on sex or type of MSKD; cost effectiveness analysis includes incremental cost-effectiveness ratio and incremental cost-utility ratio analysis using EQ-5D scores as an index (converted to 0 to 1) and total cost of an episode of care for the public payer and for the participants (using questionnaires) will be calculated

WEAKNESSES:

-there are affordances to get usual PT care after the education and usual medical group care arms; similarly- could the participants with persistent pain post usual PT care access the usual medical group care?

-how will going into the other treatment group be dealt with statistically- since outcomes are measured at 24 weeks, should a participant get usual PT care post- education (arm 1) or medical care (arm 3), how are there findings being integrated into the analysis? are they still considered education group, how will the additional interventions be controlled for?

**Review Type / Type d'évaluation:**	Reviewer 1 / Évaluateur 1
**Name of Applicant / Nom du chercheur:**	Roy, Jean-Sébastien
**Application No. / Numéro de demande:**	485725
**Agency / Agence:**	CIHR/IRSC
**Competition / Concours:**	Project Grant/Subvention Projet
**Committee / Comité:**	Clinical Investigation - B 2/Investigation clinique - B 2
**Title / Titre:**	Is a stepped model of care cost-effective compared to usual care for chronic musculoskeletal disorders? A randomized controlled trial

Budget Recommendation/Recommandation budgétaire:

Budget reasonable for timeline and RCT design with recruitment at two sites, as follows:

-0.6FTE for 2 research assistants at each of the 2 study sites

-student funding for 2 PhD trainees, justified as to each student's analysis of the data collected

-consumables include $365 000 for materials for intervention, training of personnel (MD and PTs), computers and recruitment using a company for advertisement ($10,000), each participant gets a one-time $40 stipend to cover transport costs for the in-person visit, this seems reasonable and in-line with ethics

-honoraria is included for the patient partner to release time to enable participation in the project, in-line with POR principles

-KT costs include $35 000 for open access publications (n=4), conferences, and workshops etc to share findings

**Review Type / Type d'évaluation:**	Reviewer 1 / Évaluateur 1
**Name of Applicant / Nom du chercheur:**	Roy, Jean-Sébastien
**Application No. / Numéro de demande:**	485725
**Agency / Agence:**	CIHR/IRSC
**Competition / Concours:**	Project Grant/Subvention Projet
**Committee / Comité:**	Clinical Investigation - B 2/Investigation clinique - B 2
**Title / Titre:**	Is a stepped model of care cost-effective compared to usual care for chronic musculoskeletal disorders? A randomized controlled trial

Please indicate your appraisal of the integration of sex as a biological variable as a strength, weakness, or not applicable to the proposal./Prière de sélectionner une option pour donner votre évaluation de l'intégration du sexe comme variable biologique en tant que point fort ou point faible de la proposition, ou en tant qu'élément non applicable à la proposition.

- (●) **Strength/Point fort**
- ( ) **Weakness/Point faible**
- ( ) **Not applicable/Non applicable**

Please indicate your appraisal of the integration of gender as a socio-cultural determinant of health as a strength, weakness, or not applicable to the proposal./Prière de sélectionner une option pour donner votre évaluation de l'intégration du genre comme déterminant socioculturel de la santé en tant que point fort ou point faible de la proposition, ou en tant qu'élément non applicable à la proposition.

- (●) **Strength/Point fort**
- ( ) **Weakness/Point faible**
- ( ) **Not applicable/Non applicable**

**Review Type / Type d'évaluation:**	Reviewer 1 / Évaluateur 1
**Name of Applicant / Nom du chercheur:**	Roy, Jean-Sébastien
**Application No. / Numéro de demande:**	485725
**Agency / Agence:**	CIHR/IRSC
**Competition / Concours:**	Project Grant/Subvention Projet
**Committee / Comité:**	Clinical Investigation - B 2/Investigation clinique - B 2
**Title / Titre:**	Is a stepped model of care cost-effective compared to usual care for chronic musculoskeletal disorders? A randomized controlled trial

Sex and/or Gender Considerations/Notions de sexe et/ou de genre:

Research "suggests that men and women differ in their pain responses and that stereotypical gender roles may contribute to differences in pain expression. Some of the principal outcome variables of the present project focus on participants' perception of musculoskeletal disorders and of pain: severity and intensity of pain, health-related quality of life, pain-related fear, pain catastrophizing and self-efficacy."

Sex and gender will be collected for each study participant, as well for each physiotherapist and family physician involved within the study to explore sex and gender contributions and also explore, if possible, sex and gender contribution related to patient providers interactions.

At baseline, eligibility criteria will be confirmed by a PT. To determine participants' baseline characteristics, participants will complete a questionnaire on sociodemographic (including sex at birth [male, female] and gender identity [e.g. male, female, two-spirit, gender fluid, non-binary].

**Review Type / Type d'évaluation:**	Reviewer 2 / Évaluateur 2
**Name of Applicant / Nom du chercheur:**	Roy, Jean-Sébastien
**Application No. / Numéro de demande:**	485725
**Agency / Agence:**	CIHR/IRSC
**Competition / Concours:**	Project Grant/Subvention Projet
**Committee / Comité:**	Clinical Investigation - B 2/Investigation clinique - B 2
**Title / Titre:**	Is a stepped model of care cost-effective compared to usual care for chronic musculoskeletal disorders? A randomized controlled trial

Adjudication Criteria/Critères de sélection

Initial Score/Cote Initiale: 3.8

Top/Bottom Selection/Groupe supérieur/inférieur

- ○ **Top/Groupe supérieur**
- ◉ **Bottom/Groupe inférieur**

**Review Type / Type d'évaluation:**	Reviewer 2 / Évaluateur 2
**Name of Applicant / Nom du chercheur:**	Roy, Jean-Sébastien
**Application No. / Numéro de demande:**	485725
**Agency / Agence:**	CIHR/IRSC
**Competition / Concours:**	Project Grant/Subvention Projet
**Committee / Comité:**	Clinical Investigation - B 2/Investigation clinique - B 2
**Title / Titre:**	Is a stepped model of care cost-effective compared to usual care for chronic musculoskeletal disorders? A randomized controlled trial

Summary of Application/Résumé de la demande:

Musculoskeletal disorders (MSKDs) are prevalent and the most common cause of chronic pain and disability. Converging evidence has shown that patient education plays a fundamental role in the management of MSKDs. Preliminary data generated by the applicants show that adding exercises or gait training does not provide additional benefits compared with patient education alone and that exercise does not lead to large improvement compared to education.
The applicants propose a randomized controlled trial to compare a stepped care model to the two most widely used models of care (i.e., usual medical care and usual rehabilitation care) for managing four highly prevalent types of musculoskeletal disorders. To do so, they will recruit 369 adult individuals in two research centres, will allocate them into three study groups (stepped care, medical care, or rehabilitation care), and follow up them for 24 weeks.

The primary outcome of the study is interference of pain on function (pain interference subscale - Brief Pain Inventory). Secondary outcomes will include pain intensity, health-related quality of life (HRQoL), pain-related fear / kinesiophobia, pain catastrophizing, pain self-efficacy, region-specific symptoms and functional limitations, and satisfaction with care.

The secondary objectives are to estimate the incremental cost-effectiveness ratio (ICER) of the stepped care model from the public payer's perspective, estimate the cost from the patient’s perspective, and estimate the incremental cost-utility ratio (ICUR) of the stepped care model.

The stepped care intervention will focus on patient education. Participants will take part in a self-management education program and will be offered two individual education sessions. The education program has already been implemented by the applicants. Participants will also watch six educational videos focusing on pain and function, persistent pain, stress, physical activity, sleep, and healthy eating habits. The other two interventions will be a 12-week rehabilitation program and a 12-week pharmacological pain management program.

The applicants hypothesize that the stepped care model will be at least as effective as usual medical and rehabilitation care in terms of patient-related outcomes but will lead to lower healthcare costs.
.

Strengths and Weaknesses/Forces et faiblesses:

Strengths:

Multisite clinical trial with two research units involved.

The project builds on the applicant's previous work and strong expertise in clinical trials.

The proposed research has a solid rationale and may have an immediate impact, as it has the potential to inform clinical decision making and influence healthcare policies.

The research seems to be feasible based on the applicant’s previous work.

A plan for potential non-responders to the self-management strategy (stepped care program) has been presented.

A knowledge translation plan is well discussed.

Weaknesses:

Participants of the stepped care group who will not improve after 6 weeks will be considered non-respondents and will be submitted to a rehabilitation program that will be similar to the one received by participants in the usual rehabilitation care group. It is unclear how the data of these patients who are to be considered non-respondents will be used. Information about this group of non-respondents may provide substantial information which could be crucial for the implementation of a stepped-care intervention in clinical realms.

I wondered whether participants in the rehabilitation and medical (pharmacological treatment) groups would receive information about the self-management of their condition. There is sufficient evidence showing that patient education is fundamental for treatment success. Why are these two groups prevented from this type of intervention and test whether the structured education program they propose leads to better outcomes?

It is unclear whether participants who already received other conservative (non-surgical) management strategies will be eligible for this trial. If so, what information will be collected about their previous treatments, and how will this be handled by the applicants? It is possible that individuals who tried several approaches without success may not be motivated and, therefore, may respond less to the proposed education/stepped care approach.
Notably, information about the psychological status (e.g., depression, anxiety, somatization, etc.) may be collected at baseline and during treatment to understand whether changes in these domains may predict treatment outcomes. The applicants will measure pain catastrophizing and kinesiophobia. I would advise explore whether it is feasible to collect other

information about participants' psychological status. These variables may affect patients' satisfaction with the proposed treatment (patients' satisfaction is an outcome measure of this trial).

The composition and role of the trial steering committee could be better described. What background will the member of this committee have? Timelines for each of the reported procedures (e.g., as reported by the applicants: approve the trial protocol and any amendments, monitor and supervise the trial, review relevant information from other sources, and resolve problems brought by investigators) should be included, if possible.

Only two research units will be involved in this trial, and both are n Quebec. Information about costs may be different across provinces. Therefore, the plan would benefit from including research centres in other provinces to inform policies better

.

Participants recruited in the usual medical care group program will be given opioids as "short-term, last-resorts if severe pain persists". More information should be provided about opioid management in the current trial to ensure best practices.

**Review Type / Type d'évaluation:**	Reviewer 2 / Évaluateur 2
**Name of Applicant / Nom du chercheur:**	Roy, Jean-Sébastien
**Application No. / Numéro de demande:**	485725
**Agency / Agence:**	CIHR/IRSC
**Competition / Concours:**	Project Grant/Subvention Projet
**Committee / Comité:**	Clinical Investigation - B 2/Investigation clinique - B 2
**Title / Titre:**	Is a stepped model of care cost-effective compared to usual care for chronic musculoskeletal disorders? A randomized controlled trial

Budget Recommendation/Recommandation budgétaire:

The applicants are requesting $ 910,000. The budget is well described and seems to be adequate for the proposed activities.

Please indicate your appraisal of the integration of sex as a biological variable as a strength, weakness, or not applicable to the proposal./Prière de sélectionner une option pour donner votre évaluation de l'intégration du sexe comme variable biologique en tant que point fort ou point faible de la proposition, ou en tant qu'élément non applicable à la proposition.

- (●) **Strength/Point fort**
- ( ) **Weakness/Point faible**
- ( ) **Not applicable/Non applicable**

Please indicate your appraisal of the integration of gender as a socio-cultural determinant of health as a strength, weakness, or not applicable to the proposal./Prière de sélectionner une option pour donner votre évaluation de l'intégration du genre comme déterminant socioculturel de la santé en tant que point fort ou point faible de la proposition, ou en tant qu'élément non applicable à la proposition.

- (●) **Strength/Point fort**
- ( ) **Weakness/Point faible**
- ( ) **Not applicable/Non applicable**

Sex and/or Gender Considerations/Notions de sexe et/ou de genre:

Strengths:

The applicants will collect information about sex and gender and will integrate this information into the data analysis. They will perform exploratory analyses to understand better the different trajectories of pain and pain-related variables across the study groups.

Weaknesses:

The proposal would benefit from specific sex- or gender-related hypotheses. A solid rationale for investigating sex and/or gender-related differences should be provided in the proposal.

**Review Type / Type d'évaluation:**	Reviewer 3 / Évaluateur 3
**Name of Applicant / Nom du chercheur:**	Roy, Jean-Sébastien
**Application No. / Numéro de demande:**	485725
**Agency / Agence:**	CIHR/IRSC
**Competition / Concours:**	Project Grant/Subvention Projet
**Committee / Comité:**	Clinical Investigation - B 2/Investigation clinique - B 2
**Title / Titre:**	Is a stepped model of care cost-effective compared to usual care for chronic musculoskeletal disorders? A randomized controlled trial

Adjudication Criteria/Critères de sélection

Initial Score/Cote Initiale: 3.7

Top/Bottom Selection/Groupe supérieur/inférieur

- ○ **Top/Groupe supérieur**
- ◉ **Bottom/Groupe inférieur**

**Review Type / Type d'évaluation:**	Reviewer 3 / Évaluateur 3
**Name of Applicant / Nom du chercheur:**	Roy, Jean-Sébastien
**Application No. / Numéro de demande:**	485725
**Agency / Agence:**	CIHR/IRSC
**Competition / Concours:**	Project Grant/Subvention Projet
**Committee / Comité:**	Clinical Investigation - B 2/Investigation clinique - B 2
**Title / Titre:**	Is a stepped model of care cost-effective compared to usual care for chronic musculoskeletal disorders? A randomized controlled trial

Strengths and Weaknesses/Forces et faiblesses:

Significance and Impact of Research

Strengths—Extremely prevalent problem, associated with major cost and disability. Potential to improve care, improve access to health care and reduce costs.

Weaknesses—

Approaches and Methods

Strengths—Outcome measures well described, summaries documenting appropriate measurement characteristics, MCID provided. Previous pilot work documents ability to recruit similar patients, although does not provide direct support for described interventions

Weaknesses— recruitment is via multiple mechanisms, including presentation to family medicine groups or PT, but also advertisement, and solicitation of student groups, workers etc. Given delays in obtaining care, would it be preferable to target those earlier in their course than later (potentially more responsive to interventions). Somewhat unclear how inclusion/exclusion criteria will be ascertained—some recruited through MD, some by advertising/solicitation, and assessment by PT. For example, who will review imaging (shoulder or knee), ascertainment of stenosis, infection (neck) etc. Study intake should be more clearly described. Unclear if usual medical care group, primarily medication based, is consistent with usual medical care (wouldn’t referral to physiotherapy, usually private be more typical, rather than predominant escalating medication therapy). Sample size calculated for the intervention groups as a whole. However, it is possible (?likely) that some groups will be more or less likely to respond to the interventions (neck pain vs back pain, etc)—this may make it more difficult to identify differences in intervention groups. Consideration of powering this work to analyze the patient groups (back, neck, shoulder, knee) may be beneficial . Contamination (patients using medication on their own, exercise or formal PT) seems to be likely. How will this be monitored, and how will this be handled in analysis? Cointervention also seems likely—states that this will be monitored, but no discussion of which cointerventions will be monitored and how this will be done. Very little detail is given to support the economic analysis (single sentence page 9).

Expertise, Experience and Resources

Strengths—Group has all relevant needed expertise. Successful conduct of similar research supports feasibility. Protected time for key researchers. Appropriate environment

Weaknesses—

**Review Type / Type d'évaluation:**	Reviewer 3 / Évaluateur 3
**Name of Applicant / Nom du chercheur:**	Roy, Jean-Sébastien
**Application No. / Numéro de demande:**	485725
**Agency / Agence:**	CIHR/IRSC
**Competition / Concours:**	Project Grant/Subvention Projet
**Committee / Comité:**	Clinical Investigation - B 2/Investigation clinique - B 2
**Title / Titre:**	Is a stepped model of care cost-effective compared to usual care for chronic musculoskeletal disorders? A randomized controlled trial

Budget Recommendation/Recommandation budgétaire:

Staff 385k—2 part time RA (1 in each city)-OK

Trainees 125k—2 doctoral trainees-OK

Consumables 365k—primarily to pay for FP and PT assessments and interventions, although also collaborators. Is this acceptable? At least need to document that health care system or insurance provider are not also billed.

KT 35k—OK

Please indicate your appraisal of the integration of sex as a biological variable as a strength, weakness, or not applicable to the proposal./Prière de sélectionner une option pour donner votre évaluation de l'intégration du sexe comme variable biologique en tant que point fort ou point faible de la proposition, ou en tant qu'élément non applicable à la proposition.

- ◉ **Strength/Point fort**
- ○ **Weakness/Point faible**
- ○ **Not applicable/Non applicable**

Please indicate your appraisal of the integration of gender as a socio-cultural determinant of health as a strength, weakness, or not applicable to the proposal./Prière de sélectionner une option pour donner votre évaluation de l'intégration du genre comme déterminant socioculturel de la santé en tant que point fort ou point faible de la proposition, ou en tant qu'élément non applicable à la proposition.

- ◉ **Strength/Point fort**
- ○ **Weakness/Point faible**
- ○ **Not applicable/Non applicable**

**Review Type / Type d'évaluation:**	Reviewer 3 / Évaluateur 3
**Name of Applicant / Nom du chercheur:**	Roy, Jean-Sébastien
**Application No. / Numéro de demande:**	485725
**Agency / Agence:**	CIHR/IRSC
**Competition / Concours:**	Project Grant/Subvention Projet
**Committee / Comité:**	Clinical Investigation - B 2/Investigation clinique - B 2
**Title / Titre:**	Is a stepped model of care cost-effective compared to usual care for chronic musculoskeletal disorders? A randomized controlled trial

Sex and/or Gender Considerations/Notions de sexe et/ou de genre:

both sex and gender are explicitly included in the data collection and analysis
